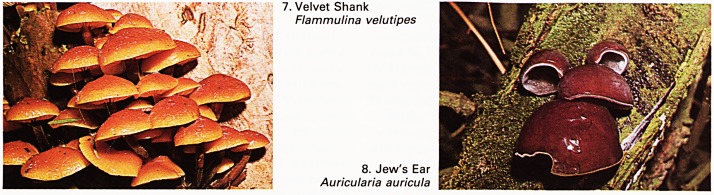# Fungi, Food, Poison and Mystery

**Published:** 1982

**Authors:** Philip Radford

**Affiliations:** General Practitioner (Rtd.), Westbury-on-Trym


### Fungi: Food, Poison and Mystery

**Philip Radford**
General Practitioner (Rtd.), Westbury-on-Trym

When we speak, loosely, of the structures we call mushrooms and toadstools, we refer to the fruiting bodies of fungi. Fungi, unlike green plants which absorb carbon dioxide from the air, obtain their nutrients by breaking down the tissues of wood, leaves, roots or, occasionally, animals, either living or dead. Essentially, fungi consist of ramifying threads or hyphae and these, together, form the mycelium; when two mycelial groups conjugate under the correct climatic conditions, then fruiting bodies are formed. It is the fruiting body which liberates the spores of the fungus. Each spore contains a single cell and one reproductive body produces them in their millions. In contrast, a seed of a green plant contains a complete embryo. Fungi play an important part in the decay of dead vegetable material; but for fungi, our forests would appear very different places.

Fungi are also important, in association, in stimulating the optimal growth of certain plants; thus, it has been shown that fungi must be present in the soil if various orchids are to grow successfully. Again, there seems no doubt that some conifer species are more healthy when they produce particular short roots whose function appears to be entirely that of fungal association; Scots pine *Pinus sylvestris* is an example here.

Fungal spores are produced in two ways. Firstly, in the Ascomycetes, the most abundant yet least conspicuous group, the spores are formed in a club-shaped body, the ascus and, when mature, these spores are shot out in their dispersal. Basidiomycetes include the most spectacular and well-known fungi; in this group, the spores mature from projections at the top and, subsequently, outside the basidial cells. These spores are just dropped when fully developed. While identification of the fruiting body must depend, in the ordinary way, on macroscopic characteristics, microscopic examination of the spores is necessary in some cases for exact classification.

Depending on the species, fungal spores may be ovoid, elliptical, polygonal, round or other different forms. In addition, spores of some species show warts or spines which may be separate or connected by lines. More simply, in the case of the larger brackets or agarics, spore prints can be obtained and will give valuable information. The fungal cap is placed on paper, gills downwards, for a few hours when a print derived from the dropped spores will have resulted. Naturally, if a black or brown print is expected, white paper is used; however, if a while spore print is likely, then coloured paper should be utilised. By means of such tests, it is usually possible to arrive at an exact identification for most of the commoner British fungal species; of course, it must be remembered that there are two or three thousand larger species which grow in Britain and, at times, more detailed historological or chemical tests are required.

A visit to any greengrocer's shop will show the ready availability of cultivated mushrooms *Agaricus bisporus* and which now feature regularly in the British diet: clearly, mushrooms are relied on to make a tasty addition to many meals. A fungal meal provides cellulose, mineral salts and small quantities of protein and carbohydrate; further, vitamins of the A, B and C groups are found in variable concentrations and, on occasions, vitamin D is present. The cultivated mushroom is eaten by most people with impunity; nevertheless, some individuals are unable to tolerate it and react with nausea, abdominal pain, sweating and tachycardia.

There are many wild fungi, however, which are both edible and delicious and it is surely foolish to ignore them should one come across a supply. The doctor living in the country may well be asked his opinion as to the value of local fungi; after all, it is known that he will have had some training in botany and is expected to understand what items will affect the body adversely. Many people regard the cultivated mushroom as a delicacy, but the field mushroom *Agaricus campestris* is much superior in flavour. The appearance of the agaric, with its pink gills and white stalk with ring, is similar to that of the cultivated mushroom; it grows in late summer and autumn in pasture fields.

Another common and edible fungus is the lawyer's wig or shaggy ink-cap *Coprinus comatus.* This species has gills which turn black and inky when over-ripe, so it should be sampled when young. Sometimes it is frequent on road verges and sports fields, where the ground has been disturbed. In the same ink-cap group is the smooth ink-cap *Coprinus atramentarius.* Like the shaggy type it is of good flavour but it reacts with alcohol, so it is not to be recommended if a glass of beer or wine is fancied as well. This interaction is the basis of the antabuse treatment of alcoholism: alcohol ingestion provokes nausea and vomiting.

A fungus which is locally common on grassland in autumn is the elegant parasol mushroom *Lepiota procera* (Plate 1). It is characterised by a scaly top to its cap, crowded white gills and a tough double ring on its long stipe. The flesh is sweeter than the lawyer's wig or the field mushroom and adds distinctiveness to any meal. The boletus family of fungi are recognisable because their spores are produced from pored tubes instead of gills. Perhaps the most renowned of these for flavour is the edible bolete or cep *Boletus edulis* (Plate 2). It grows into a large mushroom-type fungus which is found in mixed woods in autumn; it may be dried and used to flavour soups, but I much prefer it fried or grilled whole. The taste is excellent and quite unique. Usually found in association with larch *Larix decidua,* is the larch bolete *Boletus elegans.* The cuticle is sticky and yellow and the stalk is ringed; this is one of the few boletus fungi to carry a ring. Like the cep, it is of admirable flavour although, in my view, it does not rate so high in quality.

Many fungal species in the bracket form, growing horizontally from dead or partly dead wood, also have pores on the undersides of the caps yet they are mycologically distinct from the boletus group. One edible bracket is the beefsteak fungus *Fistulina hepatica* which is common on decaying oak *Quercus robur;* the flesh is red and, fancifully, meat-like. Anyway, it will eke out a frugal meal if well-cooked. A bracket fungus of preferable flavour and consistency is the sulphur polypore *Polyporus sulphureus;* otherwise known as the chicken of the woods, it sprouts in tiers, yellow and conspicuous, from decaying tree trunks. The taste is slightly acidic but it is a succulent fungus and well worth gathering for a memorable supper.

A bracket with gills instead of pores is the oyster mushroom *Pleurotus ostreatus* which is destructive to beech *Fagus sylvatica* timber. The radiating white gills, curiously, will drop lilac spores and, more than once, I have been surprised at the colour of the pattern; as oyster fungi grow throughout the year there is always something to search for to liven up the dinner table. It should be emphasised, however, that no fungus should be eaten unless it has been identified with certainty. Moreover, it is advisable, when sampling a species for the first time, to take only a small quantity, in case there is an individual idiosyncrasy. Several attractive and popular mycological identification books are now available, so it is not difficult to become familiar with the commoner edible species.

However, from the doctor's viewpoint, it is probably more important to be able to pick out the poisonous rather than the edible fungal species. The general practitioner or the hospital casualty officer are equally liable to be confronted with a case of suspected fungal poisoning, and also, if they are lucky, to be shown some of the suspicious agarics for possible confirmation. When deaths do occur, and this happens every autumn in Europe, the fatalities are caused by the death cap *Amanita phalloides.* With this species the cap is greenish and the gills white; the stalk shows a membranous, drooping ring and, at the base, is a white bag-like volva or membrane. If any fungus suggestive of this description is located in woodland, it must certainly be kept away from the cooking pot! Nevertheless, should the species be ingested, symptoms are delayed for several hours when abdominal pain, nausea and, often, diarrhoea arise. Death may be caused by eating just one the agarics; it is due to hepatic and renal failure, usually after an illness of a few days or a week. The toxin, which is heat-stable, is a polypeptide of the amanitoxin group. If a doctor, in autumn, comes across a case which features dehydration, vasomotor collapse and jaundice, it is as well to remember the possibility of poisoning by death cap.

There is no problem in the identification of the lovely fly agaric *Amanita muscaria* (Plate 3). The rich scarlet cap with white warts is spectacular on its white, ringed stalk, which shows a volva at its base; the fungus occurs in conifer and birch *Betula pendula* woods. The agaric has long been used to kill or stupefy flies and, in small quantities, it is given out to induce hallucinations in primitive societies. When eaten in larger amounts, the symptoms of poisoning are rapid in onset; salivation and lachrymation occur, with a rapid pulse of small volume and sweating. Recovery is the rule after poisoning with this species; the patients are often children who are tempted by the bright colouring of the fungus. Treatment is by intravenous atropine to combat the muscarine and choline intoxication.

Also producing hallucinations after eating are the tiny liberty caps *Psilocybe semilanceata.* Young people may hold parties where experimentation with the fungus takes place and accounts often appear in the newspapers in autumn. The cap is pointed, the gills are brown and the stalk thin and wavy; the term 'liberty' originated because of the resemblance of the cap to a popular hat worn during the French Revolution. A country practitioner could easily become involved in the treatment of affected patients. Then attractively coloured woodland fungi which cause vomiting and nausea are to be found amongst the brittle-gills. Russulas have sparse, broad and fragile gills; amongst them is a sickener *Russula emetica* with smooth, scarlet cap and white

1. Parasol Mushroom
*Lepiota procera*

2. Edible bolete or Cep
*Boletus edulis*

3. Fly Agaric
*Amanita muscaria*

4. Brittle-gill Sickener
*Russula fragilis*

5. Stinkhorn
*Phallus impudicus*

6. Common Earth-Ball
*Scleroderma aurantium*

7. Velvet Shank

*Flammulina velutipes*

8. Jew's Ear
*Auricularia auricula*

gills and stipe. It causes vomiting when eaten, except when it is well cooked; obviously the toxin is heat-labile. A fungus with similar appearance and properties is *Russula fragilis* (Plate 4) and another, found in beech woods, is *Russula mairei.* These are also said to be wholesome after heating but, personally, I would not take the risk!

Because of its phallic shape, it might be expected that the stinkhorn *Phallus impudicus* (Plate 5) could have psychological implications. Indeed, when in the egg or peridum stage, below ground and then almost odourless, it was used as an aphrodisiac in rural areas. It has been asserted that the peridium is edible and of good flavour but, as it grows upwards as a phallus, so the fly-attracting carrion-like odour makes it repellent to humans with a sense of smell. As the spores of the stinkhorn are produced inside the peridium, later to be carried upwards by the top of the phallic shaft, the species is known as a stomach fungus. One species of stomach fungus which is irritant if eaten is the common earth-ball *Scleroderma aurantium* (Plate 6); incidentally, in the past this has been sold, by fraud, as a substitute for truffles *Tuber aestivum,* although it causes nausea and vomiting. But a stomach fungus which is well worth trying is the early, white stage of the giant puff-ball *Lycoperdon giganteum:* there is hardly a better meal than this, when sliced and fried.

There seems little doubt that fungi, whether edible or poisonous for man, have diverse and interesting properties. Complex organic chemicals are elaborated, some of which have unusual physical or pharmacological characteristics. For instance, the remarkable amount of slimy mucus which covers the beautiful beech or porcelain caps *Oudemansiella mucida* suggests a highly efficient method of chemical synthesis. Again, velvet caps *Flammulina velutipes* (Plate 7) grow on dead wood in the winter months. Not only do they have a slimy surface but they can withstand severe frosting and, as a bonus, they provide a fine addition to supper on a December evening.

Also edible and attached to dead branches or trunks, normally elder *Sambucus nigra,* is the Jew's ear *Auricularia auricula* (Plate 8). The fungal cells of this species must contain an efficient anti-freeze system as heavy frosts do not appear to cause damage. If they are picked for eating, it is advisable to choose young specimens for the older ones do get rather tough and fibrous. It might be thought that if fungi are good food, they should be cultivated; however, unfortunately there has been little success in such attempts and it is only the cultivated mushroom which is grown in quantity or, indeed, can be grown for the domestic market. In general, the investigation of some fungi is only incomplete and climatic and habitat requirements are only partly understood; moreover, it could well be that certain fungal chemicals have pharmacological properties with possible medical applications which have not yet been isolated or researched. Many people are, understandably from the psychological point of view, suspicious of fungi and consider them mysterious as well as potent. It is surprising how many persons still insist that they would be afraid to eat any fungus other than the bought cultivated mushroom. But whatever may be an individual's attitude to fungi, many will admit that the fruiting bodies, particularly when seen growing in their natural settings, are amongst the most beautiful, albeit ephemeral, in the plant world, As Shelley wrote:

"And agarics, and fungi, with mildew and mould
Started like mist from the wet ground cold . . ."

## Figures and Tables

**1. 2. f1:**
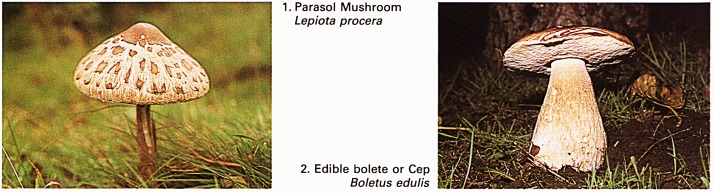


**3. 4. f2:**
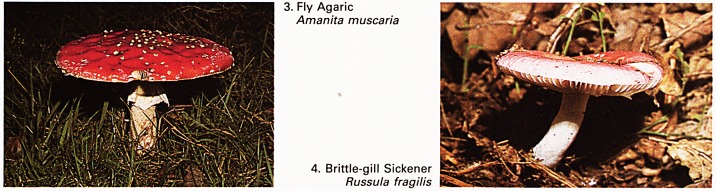


**5. 6. f3:**
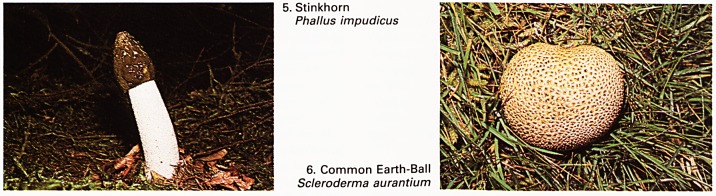


**7. 8. f4:**